# Supplementary material for: Balanced SET levels favor the correct enhancer repertoire during cell fate acquisition
Source: Nat Commun. 2023 Jun 3;14:3212. doi: 10.1038/s41467-023-39043-x (PMC10239470; doi:10.1038/s41467-023-39043-x)
Supplement: Supplementary file 1 — Supplementary Information [file 41467_2023_39043_MOESM1_ESM.pdf]

**SUPPLEMENTARY INFORMATION of the manuscript "Balanced SET levels favor the correct enhancer repertoire during cell fate acquisition" by Zaghi et al.,**

## SUPPLEMENTARY FIGURES

# Supplementary Figure 1

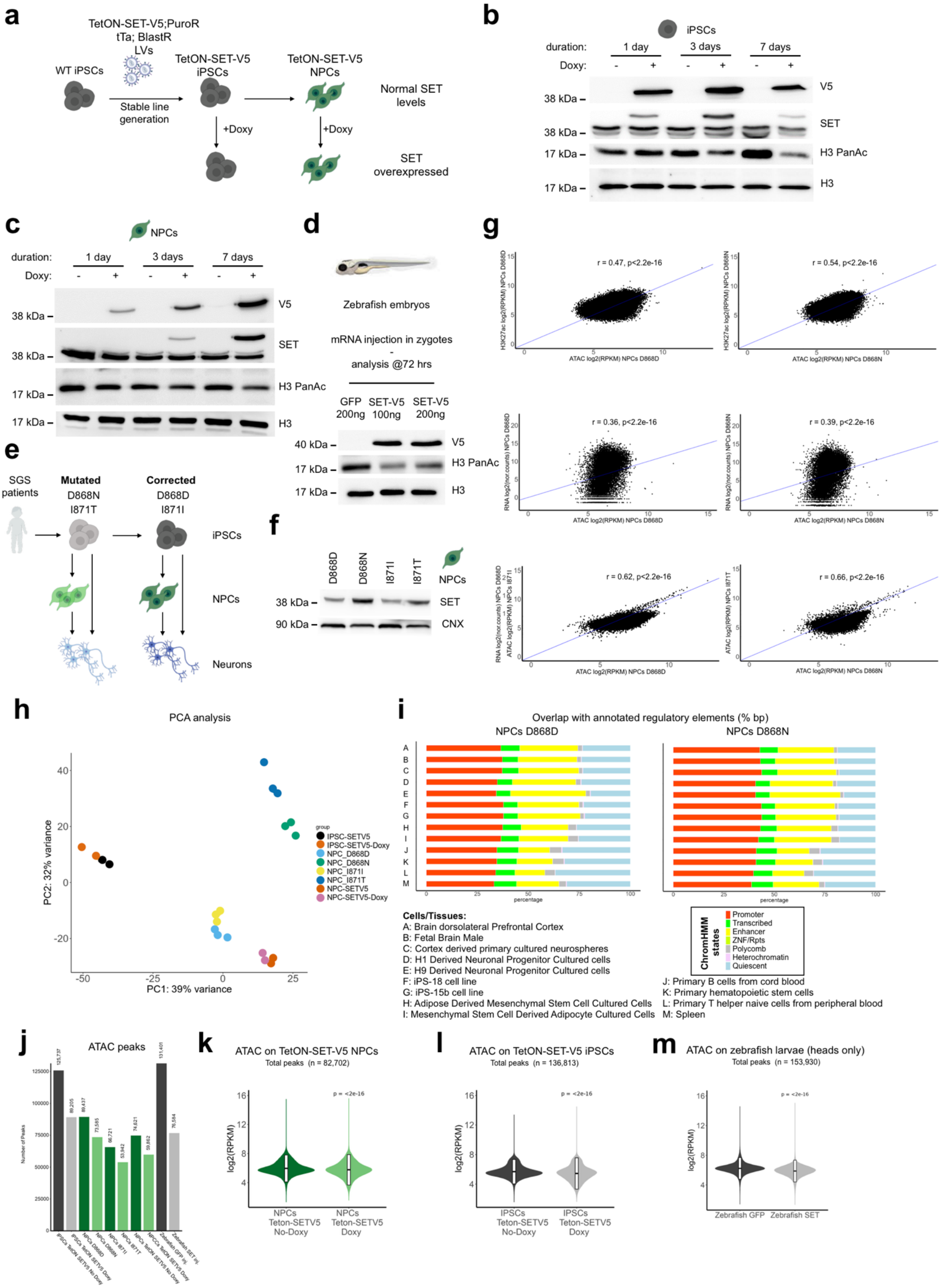

**Supplementary Fig. 1 | SET overexpression alters histone acetylation *in vitro* and *in vivo*. a**, Schematic strategy to generate inducible SET-V5 tagged overexpressing iPSCs and NPCs. Created with BioRender.com. **b, c**, Western blot for SET-V5 tagged form, total SET and H3 global acetylation levels in control (- doxy day 1, 3, 7) and overexpressing (+ doxy day 1, 3, 7) iPSCs and NPCs. H3 was used as loading control. Drawings created with BioRender.com. **d**, Western blot analysis for SET-V5 tagged form and H3 global acetylation levels in zebrafish control (200ng GFP mRNA injected) or overexpressing condition (100 ng and 200 ng SET-V5 mRNA injected). H3 was used as loading control. Drawing created with BioRender.com. **e**, Experimental scheme showing SGS patients and isogenic controls cell lines used in this work. Created with BioRender.com. **f**, Western blot analysis for SET protein expression in SGS patients derived NPCs. Calnexin (CNX) was used as loading control. Drawing created with BioRender.com. **g**, Pearson correlation between normalized ATAC-seq/H3K27ac ChIP-seq, ATAC-seq/RNA-seq signal of SGS (D868N) and control (D868D) NPCs, and normalized ATAC signal of control (D868D and I871I) and SGS (D868N & I871T) NPCs. **h**, Principal Component Analysis (PCA) of ATAC-seq samples including control (D868D, I871I, SETV5) and SET increased (D868N, I871T, SET-V5-doxy) conditions. **i**, ATAC-seq peaks of controls (D868D) and mutant (D868N) NPCs integration with available chromatin states from different tissues and cell lines. **j**, Number of ATAC-seq peaks found in each experimental condition analyzed. **k**, Violin plots of normalized signal of ATAC-seq in ATAC peaks of NPCs control (TetON-SET-V5 No-Doxy) and SET overexpressing (TetON-SET-V5 Doxy) peaks (n=82,702, Statistic two-sided Wilcoxon-test $P<2e-16$. Boxplot with 25–75th percentiles, mean, and whiskers of minima to maxima) and **l,** control (TetON-SET-V5 No-Doxy) and SET overexpressing iPSCs (TetON-SET-V5 Doxy) peaks (n=136,813, Statistic two-sided Wilcoxon-test $P<2e-16$. Boxplot with 25–75th percentiles, mean, and whiskers of minima to maxima). **m**, Violin plots of normalized signal of ATAC-seq in peaks of control (GFP mRNA injected) and SET overexpressing zebrafish (SET-V5 mRNA injected) (n=136,813, statistic two-sided Wilcoxon-test, $P<2e-16$. Boxplot with 25–75th percentiles, mean, and whiskers of minima to maxima).

## Supplementary Figure 2

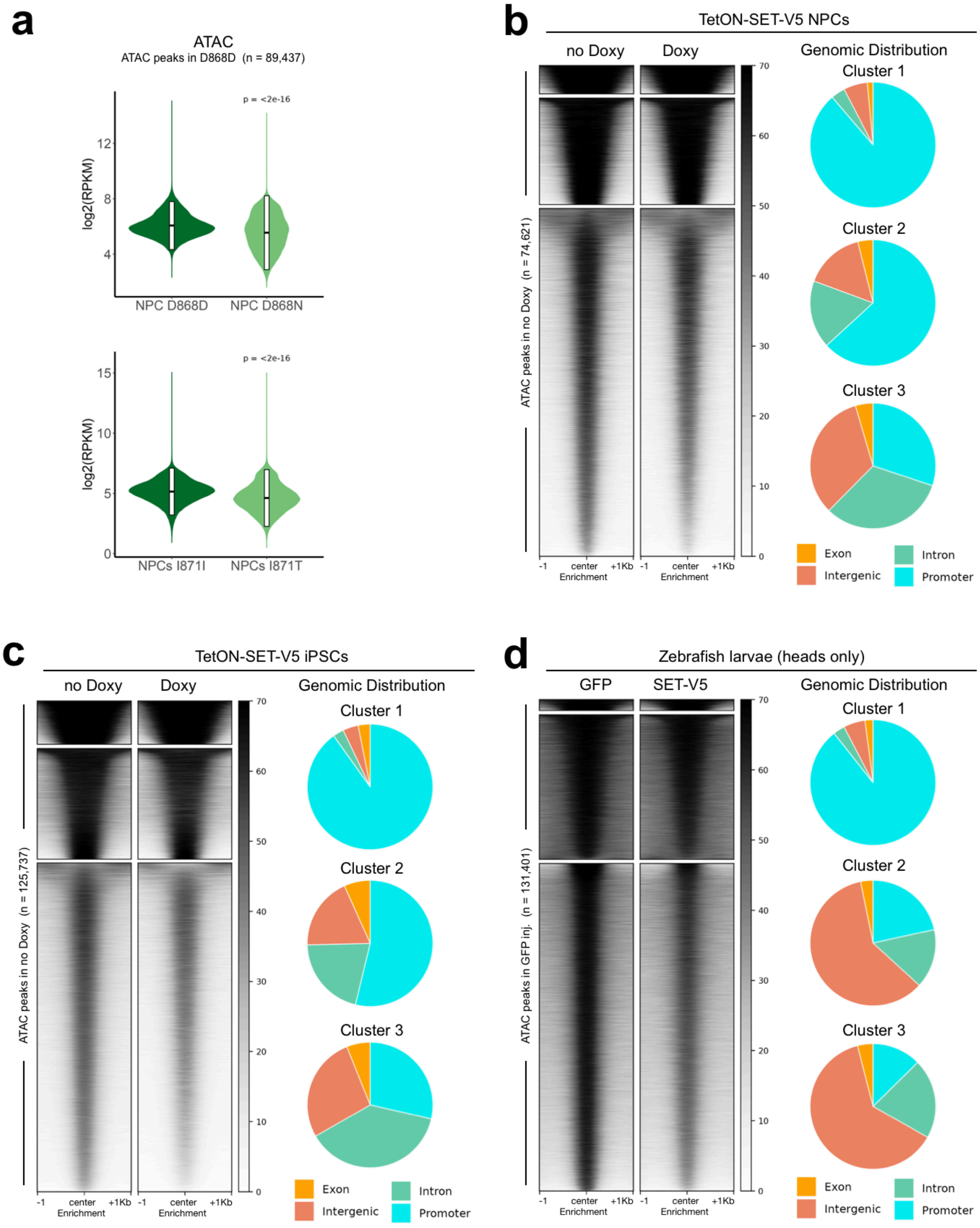


**Supplementary Fig. 2 | SET increased levels alter chromatin accessibility in different models.** **a**, Violin plots of normalized signal of ATAC-seq in peaks of NPCs control (D868D). Upper panel NPCs D868D vs NPCs D868N. Lower panel NPCs I871I NPCs I871T (n=89,437, statistic two-sided Wilcoxon-test P<2e-16. Boxplot with 25–75th percentiles, mean, and whiskers of minima to maxima) **b**, Heatmap of ATAC-seq normalized signal in control NPCs (TetON-SET-V5 No-Doxy) peaks, n=74,621. Regions are clustered using k-means (n=3), cluster1=4,165, cluster2=16,382,

cluster3=54,704. Piecharts representing the genomic distribution of regions inside the clusters. **c**, Heatmap of ATAC normalized signal in control iPSCs (TetON-SET-V5 No-Doxy) peaks, n=125,737. Regions are clustered using k-means (n=3), cluster1=11,616, cluster2=29,929, cluster3=84,192. Piecharts representing the genomic distribution of regions inside the clusters. **d**, Heatmap of ATAC normalized signal in control Zebrafish (GFP mRNA injected) peaks, n=131,401. Regions are clustered using k-means (n=3), cluster1=11,616, cluster2=29,929, cluster3=84,192. Piecharts representing the genomic distribution of regions inside the clusters.

# Supplementary Figure 3

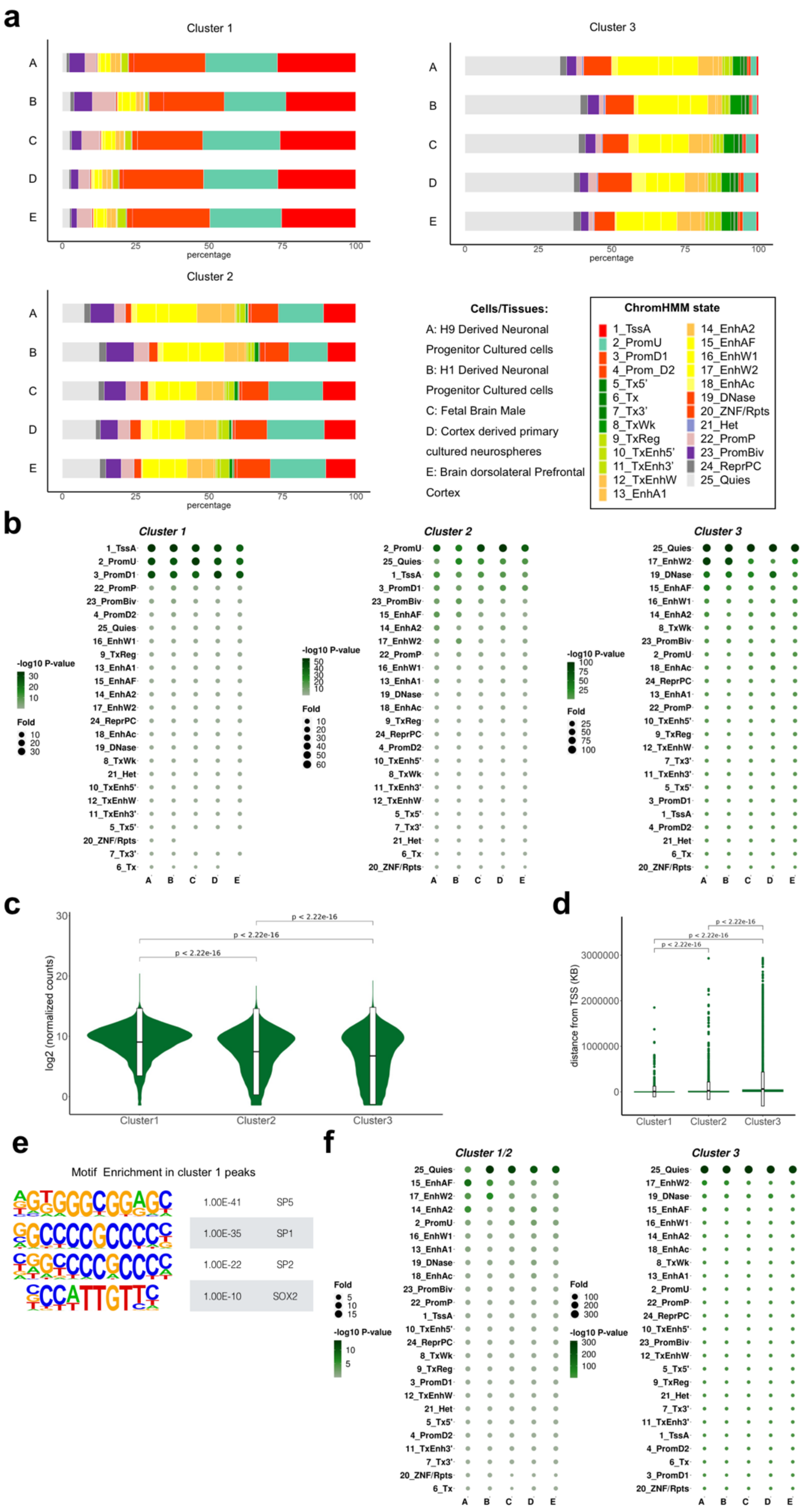

**Supplementary Fig. 3 | SET increased levels alter chromatin accessibility at distal regulatory regions.** **a**, Integration with publicly available chromatin states from different tissues and cell lines of the ATAC-seq peaks of the clusters of controls NPCs (D868D) as in **Fig. 2a**. **b**, Fold enrichment and statistical significance calculation of ChromHMM state enrichment presented in (**a**). Statistic, Hypergeometric test. **c**, Violin plot showing the average expression levels of genes associated with ATAC-seq peaks in Cluster 1,2 and 3 in control NPCs (D868D) (statistic two-sided Wilcoxon-test; Cluster1 vs Cluster2, P<2e-16; Cluster 1 vs Cluster3, P<2e-16; Cluster 2 vs Cluster3, P<2e-16, n=3 independent experiment). **d**, Boxplot showing the average distance from TSS of the nearest gene for ATAC-seq peaks inside Clusters 1,2,3 in SGS NPCs (Cluster1 n=8,033, Cluster2 n=21,201, Cluster3 n=60,203, Statistic two-sided Wilcoxon-test. Cluster1 vs Cluster2 P<2e-16; Cluster1 vs Cluster3 P<2e-16; Cluster2 vs Cluster3 P<2e-16. Boxplot with 25–75th percentiles, mean, and whiskers of minima to maxima). **e**, Homer motifs enrichment results in cluster1 of **Fig. 2a** result summary. **f**, Fold enrichment and statistical significance calculation of ChromHMM state enrichment in distal regions (intronic and intergenic) of Cluster1/2 (8426 regions) and Cluster3 (21201). Statistic two-sided Hypergeometric test.

## Supplementary Figure 4

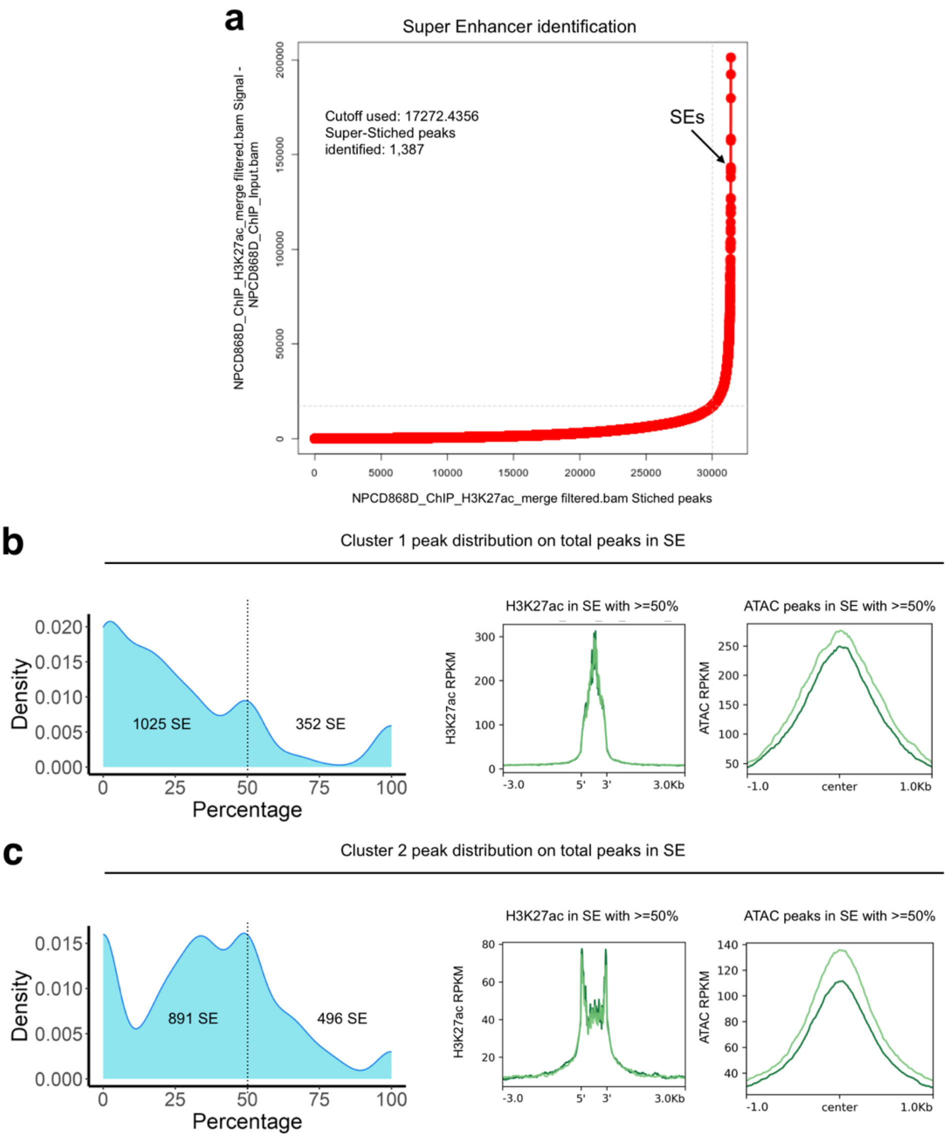


**Supplementary Fig. 4 | SET function in Superenhancers. a**, Summary plot of ROSE software results showing the ratio between H3K27ac ChIP-seq signal in control (D868D) NPCs and its respective input used to identify the Superenhancers (SEs) value cutoff starting from NPCs (D868D) controls peaks, n=89,437. **b**, Right, density plot of SE based on the percentage of cluster 1 ATAC peaks present inside each SE. Left, density plots showing H3K27ac ChIP-seq and ATAC-seq normalized signal inside SEs with more than 50% of associated ATAC peaks belonging to cluster 1 of **Fig. 2a**, n=352. **c**, Right, density plot of SE based on the percentage of cluster 2 ATAC peaks present inside each SE. Left, density plots showing H3K27ac ChIP-seq and ATAC-seq normalized signal inside SEs with more than 50% of associated ATAC peaks belonging to cluster 2 of **Fig. 2a**, n=496.

## Supplementary Figure 5

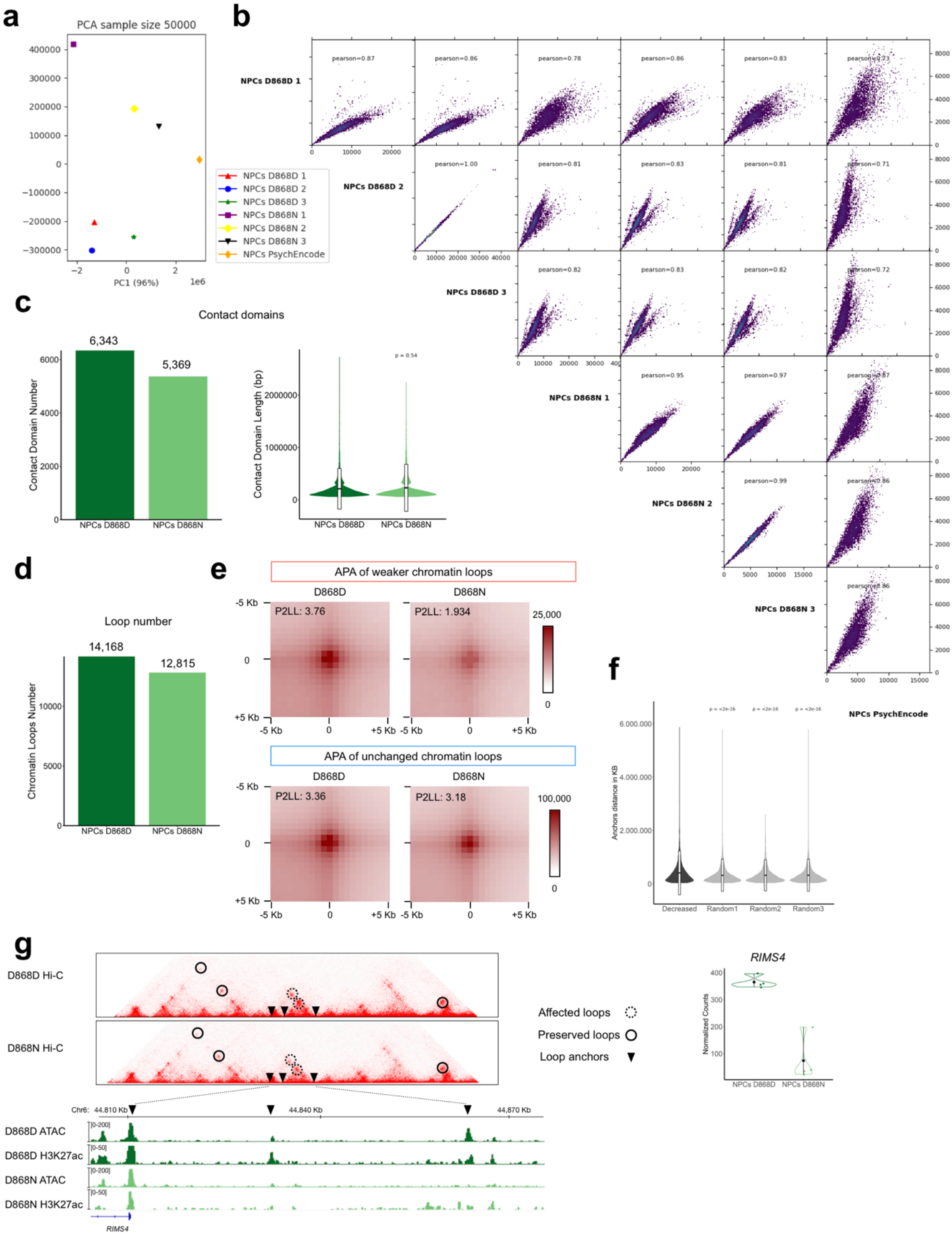


**Supplementary Fig. 5 | Chromatin structure is partially altered in SGS NPCs. a**, PCA of NPCs Hi-C samples together with a NPC PsychEncode sample. **b**, Pearson correlation matrix of NPCs Hi-C samples together with a NPC PsychEncode sample. **c**, Number (D868D n=6,343, D868N =5,369) and length plots of contact domain in NPCs control (D868D) and mutant (D868N). The mean value

plus/minus the standard deviation is plotted in the violin plot (statistic two-sided Wilcoxon-test, P<2e-16. Boxplot with 25–75th percentiles, mean, and whiskers of minima to maxima). **d**, Bar plot of loop number (D868D n=14,168, D868N =12,815). **e**, APA plots of decreased strength chromatin loops (n=2,978) and unchanged (n=11,190) in control (D868D) and mutant (D868N) NPCs. **f,** Violin plot of loop length comparing the length of decreased strength loop (n=2,978) and 3 subsamples containing the same loop number randomly chosen between the unchanged loops. The mean value plus/minus the standard deviation is plotted in the violin plot. Statistic, Wilcoxon-test. **g**, Left, genomic locus of *RIMS4* gene showing interaction frequencies maps with underlined chromatin loops and genome browser of ATAC-seq and H3K27ac ChIP-seq of control (D868D) and mutant (D868N). Right, Mean RNA expression level of *RIMS4* in control (D868D) and mutant (D868N) NPCs is shown.

# Supplementary Figure 6

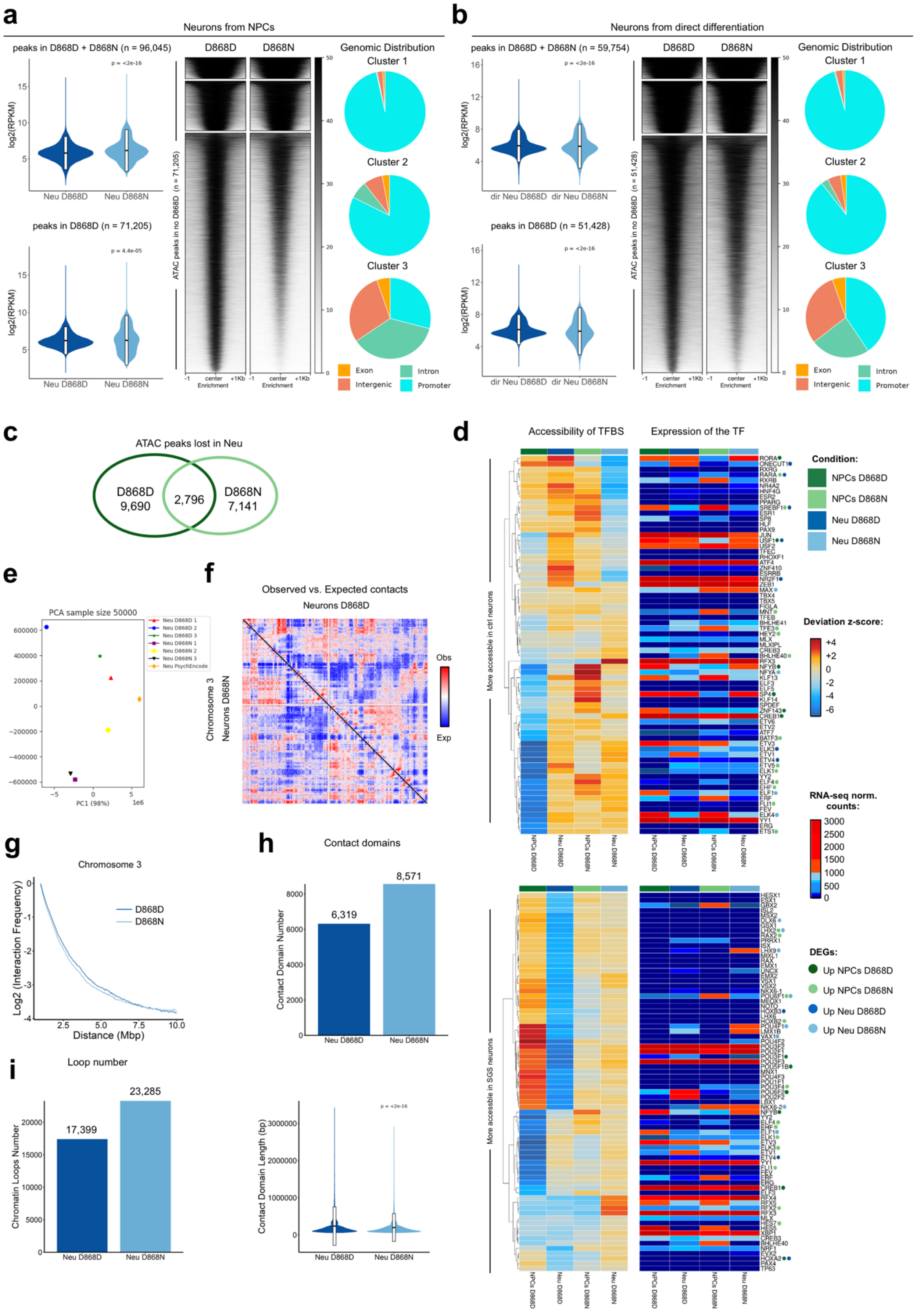

**Supplementary Fig. 6 | SGS neurons present a differential chromatin signature during development. a**, Violin plots of ATAC-seq normalized signal in NPC-derived neurons on all peaks found (control (D868D) + SGS (D868N) (top, n=96,045, statistic two-sided Wilcoxon-test, P<2-e16. Boxplot with 25–75th percentiles, mean, and whiskers of minima to maxima) and on control (D868D) only peaks (bottom, n=71,205, statistic two-sided Wilcoxon-test, P=4.4e-05. Boxplot with 25–75th percentiles, mean, and whiskers of minima to maxima). Heatmap with clusters (see methods) of ATAC-seq normalized signal in control (D868D) NPC-derived neurons peaks, n=71,205. Cluster1=4,817, cluster2=11,329, cluster3=55,060. Pie charts representing the genomic distribution of regions inside the clusters. **b**, Violin plots of ATAC-seq normalized signal in iPSC-derived neurons on all peaks (control (D868D) + SGS (D868N) (top, n=59,754, statistic two-sided Wilcoxon-test, P<2-e16. Boxplot with 25–75th percentiles, mean, and whiskers of minima to maxima) and on control (D868D) only peaks (bottom, n=51,428, statistic two-sided Wilcoxon-test, P=4.4e-05. Boxplot with 25–75th percentiles, mean, and whiskers of minima to maxima). Heatmap with clusters (see methods) of ATAC normalized signal in control (D868D) iPSC-derived neurons peaks, n=51,428. Cluster1=3,486, cluster2=8,634, cluster3=39,308. Pie charts representing the genomic distribution of regions inside the clusters. **c**, Venn diagram of overlapping peaks significantly downregulated in NPC-derived neurons during neural development in control (D868D) and mutant (D868N). **d**, Paired Heatmaps of TFBS deviations z-score and relative normalized counts expression level of the correspondent gene (upper part: TFBS more accessible in control neurons; lower part: TFBS more accessible in SGS neuron. Colored points next to TF name indicate if they are upregulated as indicated in legend. **e**, PCA of Hi-C neurons samples together with a PsychEncode sample. **f**, Normalized observed/expected interaction frequency map (Chr3) comparing control (D868D) and mutant (D868N) neurons. **g**, Summary plot of interaction frequency value and the contact distance between bins at 50kb resolution comparing control (D868D) and mutant (D868N) neurons. **h**, Number (top, D868D n=6,319, D868N =8,571) and length plots (bottom) of contact domain in control (D868D) and mutant (D868N) neurons (statistic two-sided Wilcoxon-test, P<2e-16. Boxplot with 25–75th percentiles, mean, and whiskers of minima to maxima). **i**, Bar plot of loop number (D868D n=17,399, D868N =23,285).

# Supplementary Figure 7

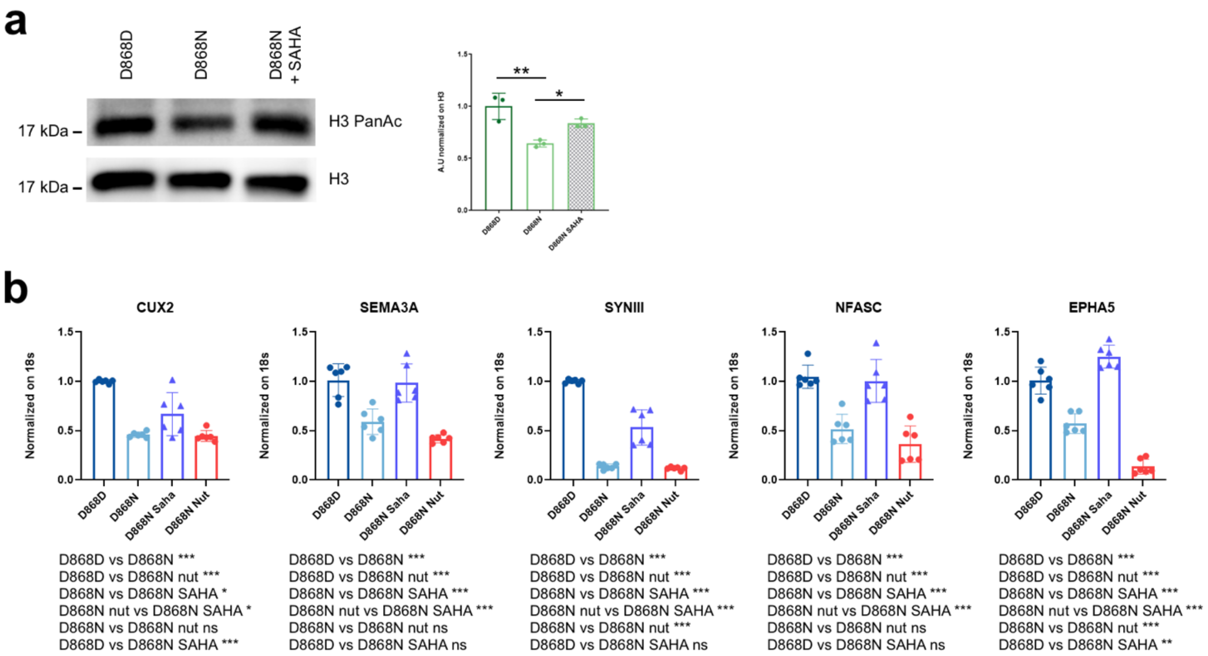


**Supplementary Fig. 7 | HDACs inhibition partially rescues SGS phenotype. a**, Western blot for H3PanAc levels on NPCs control (D868D), mutant (D868N) and mutant treated with SAHA and quantification. H3 was used as a loading control (statistic, one-way-ANOVA and one-sided Tuckey multiple comparison test, D868DvsD868N, ** P=0.0037, D868DvsD868N SAHA, ns, P=0.1017, D868N vs D868N SAHA, * P=0.0545, n=3 independent experiments. Data are presented as mean values +/- SEM). **b**, Real-time quantitative PCR (RT-qPCR) for neuronal gene levels in NPC-derived neurons control (D868D), mutant (D868N) and mutant treated with SAHA or Nutlin. (*CUX2*, statistic one-way-ANOVA and one-sided Tuckey multiple comparison test, D868DvsD868N, *** P<0.0001, D868DvsD868N SAHA, *** P=0.0003, D868DvsD868N Nutlin, *** P<0.0001, D868NvsD868N SAHA, * P=0.022, D868NvsD868N Nutlin, P= 0.9971, D868N SAHAvsD868N Nutlin, * P=0.01473, (*SEMA3A,* statistic one-way-ANOVA and one-sided Tuckey multiple comparison test, D868DvsD868N, *** P=0.0003, D868DvsD868N SAHA, P=0.9842, D868DvsD868N Nutlin, *** P<0.0001, D868NvsD868N SAHA, *** P=0.0008, D868NvsD868N Nutlin, P= 0.1977, D868N SAHAvsD868N Nutlin, *** P<0.0001), (*SYNIII*, statistic one-way-ANOVA and one-sided Tuckey multiple comparison test, D868DvsD868N, *** P<0.0001, D868DvsD868N SAHA, *** P<0.0001, D868DvsD868N Nutlin, *** P<0.0001, D868NvsD868N SAHA, *** P<0.0001, D868NvsD868N Nutlin, P= 0.9858, D868N SAHAvsD868N Nutlin, *** P<0.0001), (*NFASC*, statistic one-way-ANOVA and one-sided Tuckey multiple comparison test, D868DvsD868N, *** P=0.0002, D868DvsD868N SAHA, P=9695, D868DvsD868N Nutlin, *** P<0.0001, D868NvsD868N SAHA, *** P=0.0005, D868NvsD868N Nutlin, P= 0.4216, D868N SAHAvsD868N Nutlin, *** P<0.0001),

(*EPHA5*, statistic one-way-ANOVA and one-sided Tuckey multiple comparison test, D868DvsD868N, *** P<0.0001, D868DvsD868N SAHA, P=0.0052, D868DvsD868N Nutlin, *** P<0.0001, D868NvsD868N SAHA, *** P<0.0001, D868NvsD868N Nutlin, *** P< 0.0001, D868N SAHAvsD868N Nutlin, *** P<0.0001). N=6 independent experiments. Data are presented as mean values +/- SEM).

# Supplementary Figure 8

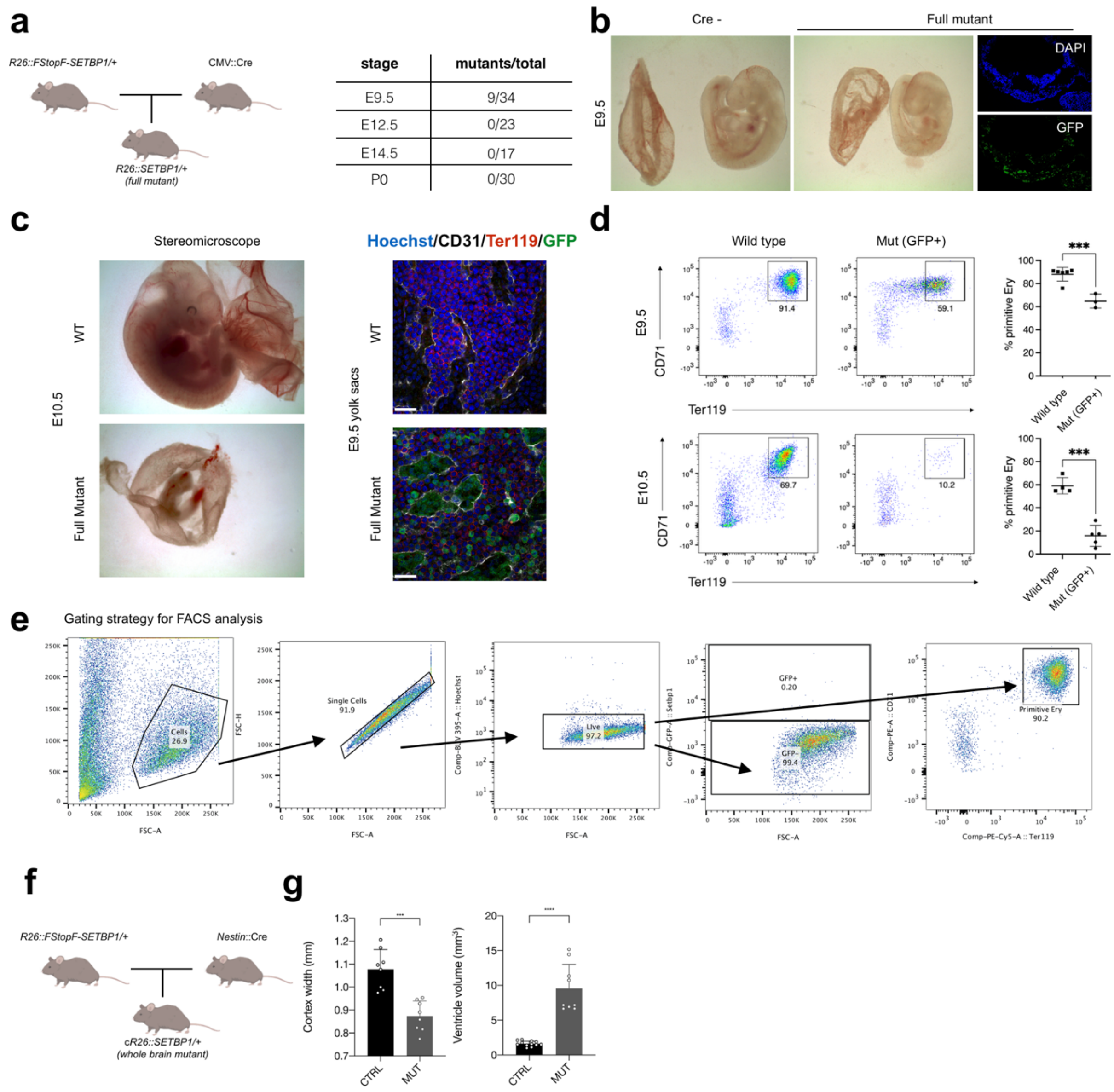


**Supplementary Fig. 8 | Whole body SETBP1 overexpression is lethal. a**, Schematic representation of genetic crossing of CMV::Cre and *Rosa26-LoxP-STOP-LoxP-hSETBP1$^{G870S}$* mice to obtain full mutants, and summary table of survival proportion at different time point. Drawings created with BioRender.com. **b**, Brightfield images of control (Cre-) and full mutant (CMV::Cre+) embryos at E9.5. On the right, immunofluorescence staining for GFP in mutant embryos, show correct expression of the exogenous cassette. **c**, Left, brightfield images of control and full mutant embryos at E10.5. Panels on the right show whole mount confocal images of yolk sacs of E9.5 embryos of the same genotypes, immunostained with Hoechst, CD31 (vasculature), Ter119 (erythrocytes) and GFP. Mutant yolk sacs show a reduced number of erythrocytes within the yolk sac vascular plexus. Bars = 30 µm. **d**, Flow cytometry analysis of E9.5 (top) and E10.5 (bottom) control and full mutant yolk

sacs, stained with CD71 and Ter119 antibodies. Dead cells were excluded by Hoechst 33258 dye incorporation. The percentage of double positive CD71+ Ter119+ cells was evaluated within live cells for control yolk sacs and within live GFP+ cells for mutant yolk sacs, and is quantified in the graphs on the right (E9.5, statistic two-sided t-test, *** P< 0.0001, n=6 for wild type and n=3 for GFP+ independent embryos; E10.5, statistic t-test, *** P< 0.0001, n=4 for wild type and n=5 for GFP+ independent embryos. Data are presented as mean values +/- SEM). **e,** Gating strategy for FACS analysis in E9.5 and E10.5 embryos on control (CRE+) and mutant (CRE+). Cells were first filtered to exclude doublets and dead cells. Then live cells were first checked for GFP expression to confirm the correct genotype and then the percentage of primary endothelium cells was determined using Ter119 and CD71 markers. **f**, Schematic representation of genetic crossing of Nestin::Cre and *Rosa26-LoxP-STOP-LoxP-hSETBP1*$^{G870S}$ mice to obtain whole brain specific mutants. **g**, Bar plots of cortex width and ventricle volume of control and brain mutant mice (cortex width, statistic two-sided t-test, Control vs. Mutant, *** P<0.001, n=8 for CTRL and n=8 for MUT independent animals; ventricle volume, Control vs. Mutant, *** P<0.001, n=12 for CTRL and n=9 for MUT independent animals. Data are presented as mean values +/- SEM).

## Supplementary Figure 9

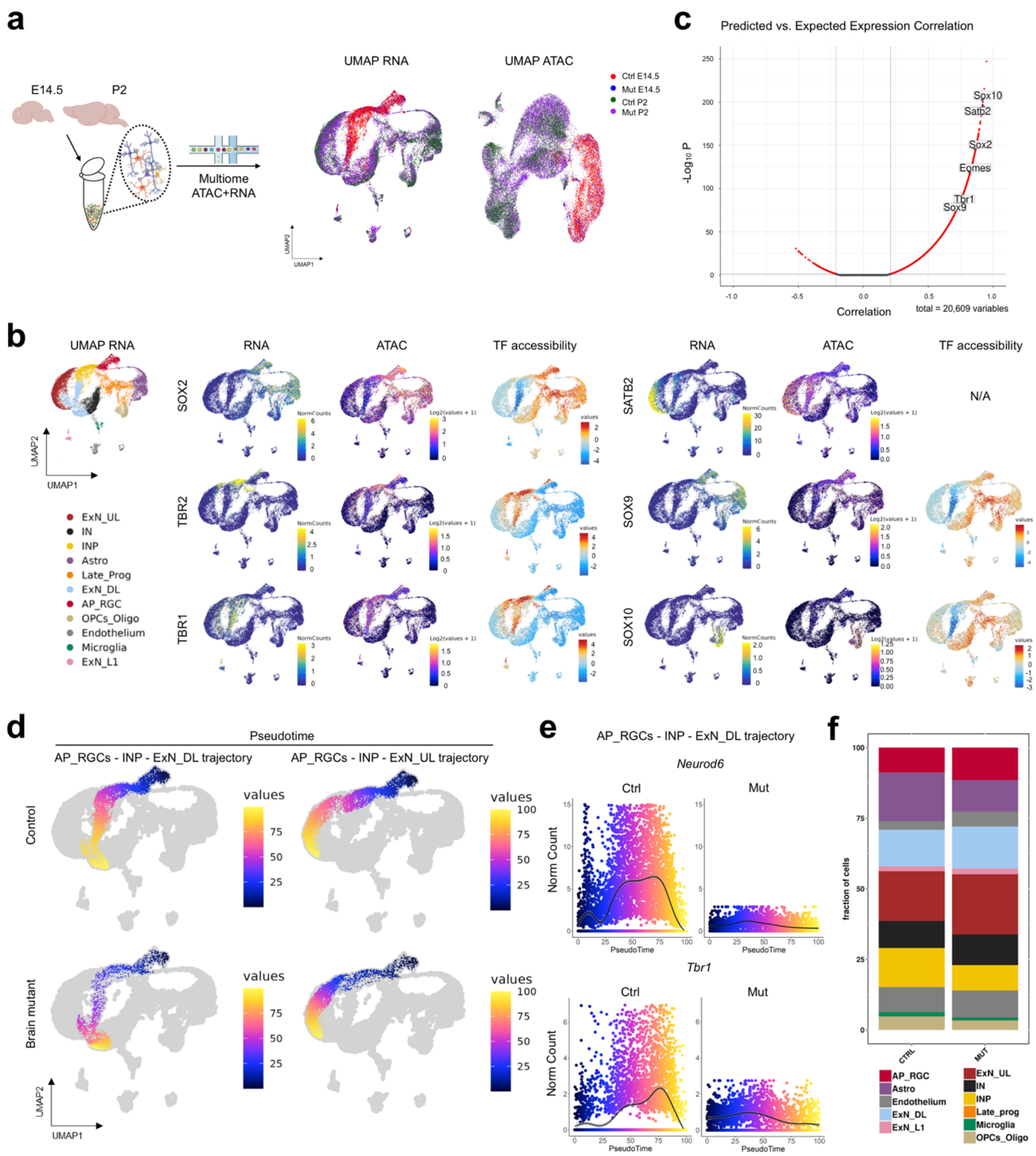


**Supplementary Fig. 9 | Brain SETBP1 overexpression alters chromatin dynamics. a**, Left, Schematic representation of scMultiome experiment on E14.5 and P2 cortices. Created with BioRender.com. Right, UMAP plots based on RNA (left) and ATAC (right) datasets with cells colored by sample. **b**, RNA UMAP feature plot showing the expression levels, accessibility predicted gene activity and TFBS accessibility level of typical neural cluster markers (SOX2, TBR2, TBR1, SATB2, SOX10, SOX9). **c**, Volcano plot showing the correlation between predicted expression based on ATAC-seq and actual gene expression for scRNA-seq data. Pearson correlation, Cut-off: P-adjusted < 0.05, Correlation > 0.10, Correlation < 0.10. **d**, RNA dataset based UMAP plots with ExN_DL and ExN_UL differentiation trajectory-associated cells colored by their pseudotime value

using control and brain mutant cells. **e**, Scatter plots showing the expression levels of *Neurod6* and *Tbr1* along AP_RGCs -> ExN_DL pseudotime trajectory. **f**, Stacked boxplot showing the normalized proportion of cells associated to each different cluster in the control and brain mutant genotype.

# Supplementary Figure 10

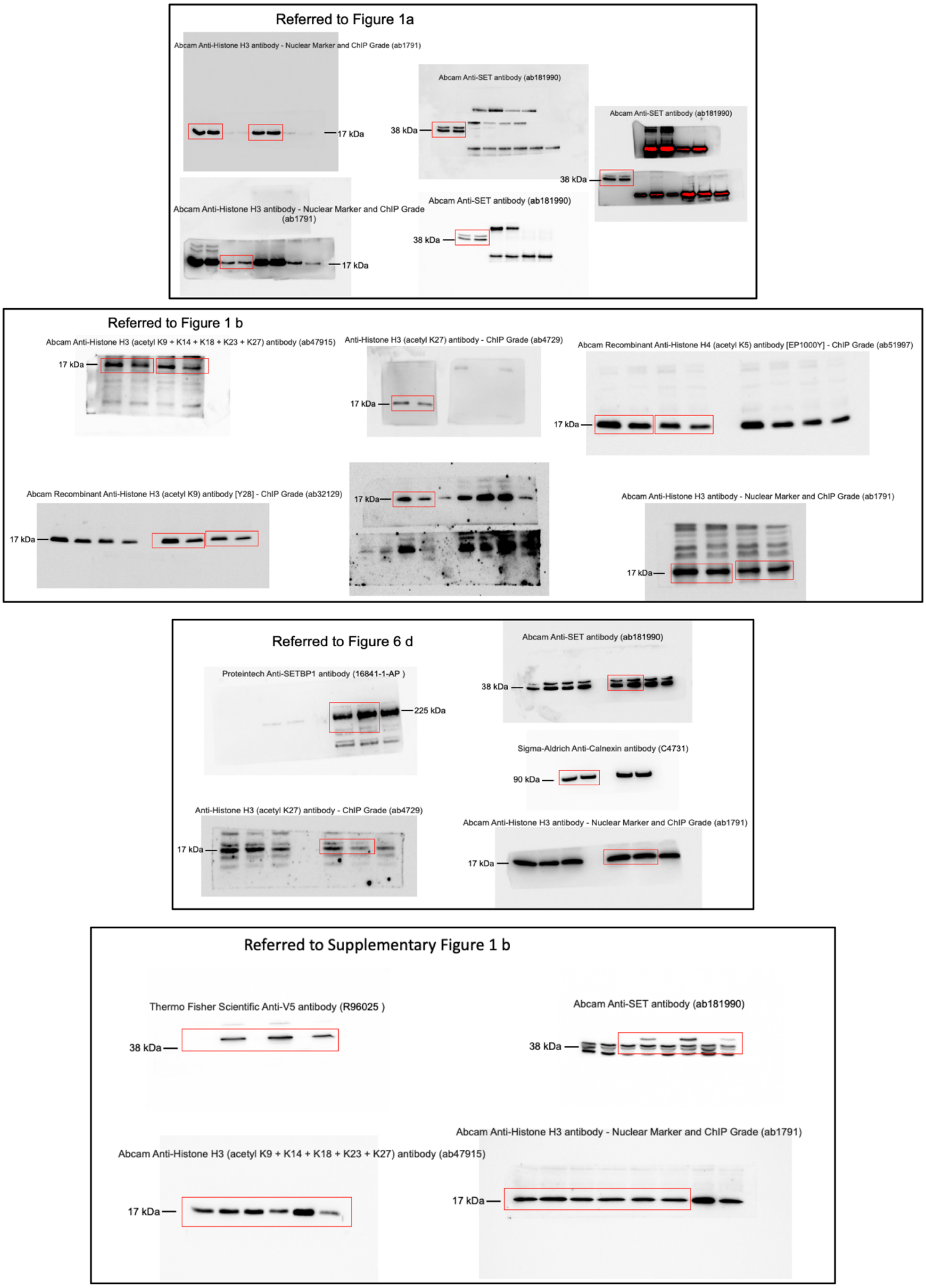


**Supplementary Fig. 10 | Uncropped Blots referred to Fig. 1a, b, Fig. 6d, Supplementary Fig. 1b.**

## Supplementary Figure 11

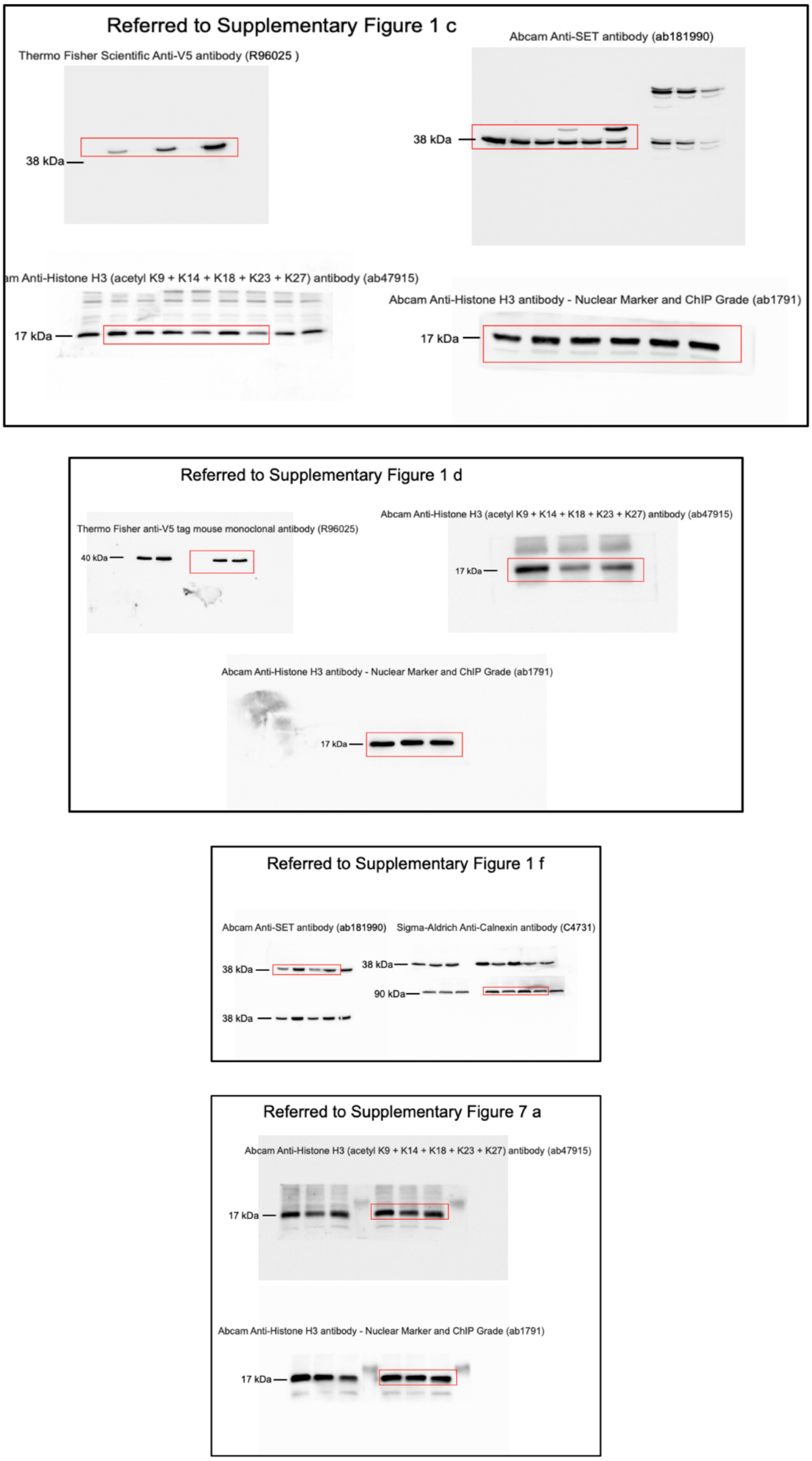


**Supplementary Fig. 11 | Uncropped Blots referred to Supplementary Fig. 1c, d, f, Supplementary Fig. 7b.**

**SUPPLEMENTARY TABLES**

**Supplementary Table 1**

| NAME | SPECIES | CATALOG | APPLICATION | DILUTION |
|---|---|---|---|---|
| SOX2 | Mouse | R&D<br>ab59776 | IF | "1:200" |
| TBR2 | <br>Rabbit | Abcam<br>ab23345 | IF | "1:200" |
| GFP | Chicken | ThermoFisher Scientific A10262 | IF | "1:1000" |
| GFP | Rabbit | Thermo-Fisher<br>A11122 | IF | "1:500" |
| CALNEXIN | Rabbit | Sigma-Aldrich<br>C4731 | WB | 1:2000, 5% MILK |
| SETBP1 | Rabbit | Proteintech<br>16841-1-AP | WB | 1:500, 5% MILK |
| SET | Rabbit | Abcam<br>ab181990 | WB/IP/ChIP | 1:500, 5% MILK,<br>ChIP/IP 5 micrograms |
| H3 | Rabbit | Abcam<br>ab1791 | WB/IP | 1:2000, 5% MILK,<br>IP 5 micrograms |
| H3K27ac | Rabbit | Diagenode<br>C15410196 | ChIP | 5 micrograms |
| H3K27ac | Rabbit | Abcam<br>Ab4729 | WB | 1:500, 5% MILK |
| H3PanAC | Rabbit | Abcam<br>ab47915 | WB | 1:500, 5% MILK |
| H3K9ac | Rabbit | Abcam<br>ab32129 | WB | 1:500, 5% MILK |
| H4K5ac | Rabbit | Abcam<br>ab51997 | WB | 1:1000, 5% MILK |
| RFP | Rabbit | Voden | IF | "1:400" |

| | | | | |
|---|---|---|---|---|
| | | PM005 | | |
| DCX | Rabbit | Abcam<br>ab18723 | IF | "1:500" |
| CD31 | Goat | R&D Systems<br>AF3628 | IF | "1:400" |
| TER119 | Rat | BioLegend<br>116241 | IF | "1:400" |
| CD71-PE | Rat | BioLegend<br>113808 | FACS | "1:100" |
| TER119-PE-Cy5 | Rat | BioLegend<br>116210 | FACS | "1:100" |
| 488 | Mouse | Thermo-Fisher<br>A11039 | IF | "1:1000" |
| 488 | Rabbit | Thermo-Fisher<br>A21206 | IF | "1:1000" |
| 488 | Rat | Thermo-Fisher<br>A21208 | IF | "1:1000" |
| 546 | Rabbit | Thermo-Fisher<br>A10040 | IF | "1:1000" |
| 555 | Rabbit | Thermo-Fisher<br>A31752 | IF | "1:1000" |
| 647 | Goat | Thermo-Fisher<br>A21447 | IF | "1:1000" |
| HOECHST | | SIGMA-Aldrich<br>33258 | | "1:1000" |
| | Goat | DAKO | WB | 1:10000, 5% MILK |

| | | | | |
|---|---|---|---|---|
| **HRP-MOUSE** | | **P044701-2** | | |
| **HRP-RABBIT** | **Goat** | **DAKO**<br>**P044801-2** | **WB** | **1:10000, 5% MILK** |

**Supplementary Table 1: List of antibodies.**

Complete recap of antibodies used in this work.

**Supplementary Table 2**

| | |
|---|---|
| 18S_Fw | 5'-GGTGAAATTCTTGGACCGGC-3' |
| 18S_Rev | 5'-GACTTTGGTTTCCCGGAAGC-3' |
| CUX2_Fw | 5'-TGTCGGATGAGCAGAATGTA-3' |
| CUX2_Rev | 5'-GCGTCGTCTGAGCCTGTCTC-3' |
| SEMA3A_Fw | 5'-GCCTTATCAAGGAAGAGTCC-3' |
| SEMA3A_Rev | 5'-GCAAAGGTTATAACATCATC-3' |
| SYNIII_Fw | 5'-ATCGGCCTGCAGTATGGAGG-3' |
| SYNIII_Rev | 5'-ATGGTTGGGGAAAAAATGTTT-3' |
| NFASC_Fw | 5'-GTGGCATGGACCTCCTGCTG-3' |
| NFASC_Rev | 5'-TCAAACTTGGCCTTATCAGA-3' |
| EPHA5_Fw | 5'-CTCACAGTTATACCCATGAG-3' |
| EPHA5_Rev | 5'-CCATTCCAGAAAGACACTAG-3' |

**Supplementary Table 2: List of oligonucleotides.**

Complete recap of primers used in this work.
