# Supplementary material for: Balanced SET levels favor the correct enhancer repertoire during cell fate acquisition
Source: Nat Commun. 2023 Jun 3;14:3212. doi: 10.1038/s41467-023-39043-x (PMC10239470; doi:10.1038/s41467-023-39043-x)
Supplement: Supplementary file 3 — Description of Additional Supplementary Files [file 41467_2023_39043_MOESM3_ESM.pdf]

Description of Additional Supplementary Files

Supplementary Data 1: H3K27ac ChIP-seq in NPCs (Fig. 1d).

Complete list of H3K27ac statistically significant ChIP-seq peaks found in NPCs D868D.

Supplementary Data 2: ATAC-seq in SGS NPCs and SET overexpressing models (fig. 1e-i, Extended data Fig. 1i-l, Supplementary Fig. 2a).

Complete list of significant ATAC-seq regions found in IPSCs, NPCs and Zebrafish model. Each sheet contains a unique peak list associated with each experimental condition.

Supplementary Data 3: ATAC-seq K-means clusters for differential accessibility analysis (Fig. 2a, b, Supplementary Fig. 2b-d).

Complete list of genomic regions associated to each cluster presented in Fig. 2a, b, Extended data Fig. 2b-d together with their positional annotation. The list of regions used for longitudinal analysis from PSCs to NPCs is also present.

Supplementary Data 4: ChromHMM state enrichment results for SGS NPCs clusters & TFBS enrichment analysis results on ATAC-seq clusters identified using accessible regions in D868D NPCs (Fig. 2c, Supplementary Fig. 3a,b,e,f).

TFBS motifs identified inside ATAC-seq clusters presented in Fig. 2a,b.

Supplementary Data 5: Super-enhancers in D868D NPCs (Fig. 2d, Supplementary Fig. 4a).

The full list of regions identified as SE using H3K27ac positive regions in D868D NPCs.

Supplementary Data 6: D868 NPCs Hi-C maps quality metric (Fig. 3a-c, Supplementary Fig. 5a, b).

Summary reports of the quality metrics of the single Hi-C replicates and the merged maps of D868D and D868N NPCs.

Supplementary Data 7: D868 NPC contact domains and loop list (Supplementary Fig. 5c,d).

Full list of chromatin loops and contact domains identified in NPCs D868D and D868N.

Supplementary Data 8: Contact analysis of D868D NPC loop anchors and associated genes (Fig. 3d-i, Supplementary Fig. 5e,f).

Complete lists of chromatin loops with increased, decreased and unchanged contact strength between NPCs D868D and D868N. The list of genes proximal (<10kb) to loops showing a decrease contact strength and the associated functional enrichment analysis (GO) is also present.

Supplementary Data 9: ATAC-seq in NPCs and iPSCs derived D868 neurons (Fig. 4a-c, Supplementary Fig. 6a-d).

Table containing all ATAC-seq statistically significant regions found in both NPC- and iPSC-derived neurons, regions with differential accessibility between NPCs and the neurons in D868D and D868N lines and the complete result of TFBS motifs accessibility analysis performed with ChromVAR.

Supplementary Data 10: Quality metrics of Hi-C maps of D868 NPC-derived neurons (Supplementary Fig. 6e-g).

Summary reports of quality metrics of the single Hi-C replicates and the merged maps of D868D and D868N NPC-derived neurons.

Supplementary Data 11: Chromatin loop contact strength changes during neural differentiation (Fig. 4d, e, Supplementary Fig. 6h, i)

Table contains the full list of loops and contact domains identified in NPCs derived neurons D868D and D868N. The summary of the loop contact strength during neuronal differentiation is present for both genotypes analyzed together with the loop anchors comparison involving loops gaining contact strength during differentiation.

Supplementary Data 12: Enhancer-promoter analysis using Hi-C and ATAC-seq data (Fig. 4f-h).

Subsets of genes associated with regions gaining accessibility in D868D and D868N NPC-derived neurons and the resulting functional enrichment analysis (GO).

Supplementary Data 13: RNA-seq analysis on NPC-derived neurons (Fig.4i)

Full RNA-seq analysis results with normalized counts and differential expressed genes of NPC-derived neurons and NPCs from GSE171266.

Supplementary Data 14: Single-cell multiome experimental summary (Fig. 6e-g, Supplementary Fig. 9a-e).

List of all single cell-associated barcodes and quality measures for both RNA and ATAC dataset with associated UMAP coordinates and the relative clusters. ATAC-seq significant peaks called for each cluster are also present for both genotypes.

Supplementary Data 15: Differential gene expression analysis in multiome clusters (Fig. 6h-j, Supplementary Fig. 9j).

Summary of the results of the differential gene expression analysis performed between control and mutant cells in each cluster.

Supplementary Data 16: Association of ATAC peaks to each gene in multiome clusters (Fig. 6h-j).

Results of ATAC peaks to gene association analysis for each cluster.
