# Supplementary material for: Balanced SET levels favor the correct enhancer repertoire during cell fate acquisition
Source: Nat Commun. 2023 Jun 3;14:3212. doi: 10.1038/s41467-023-39043-x (PMC10239470; doi:10.1038/s41467-023-39043-x)
Supplement: Supplementary file 20 — Reporting Summary [file 41467_2023_39043_MOESM20_ESM.pdf]

nature portfolio

Corresponding author(s): Sessa Alessandrio

Last updated by author(s): Mar 27, 2023

Reporting Summary

Nature Portfolio wishes to improve the reproducibility of the work that we publish. This form provides structure for consistency and transparency in reporting. For further information on Nature Portfolio policies, see our Editorial Policies and the Editorial Policy Checklist.

Statistics

For all statistical analyses, confirm that the following items are present in the figure legend, table legend, main text, or Methods section.

n/a	Confirmed	
☐	☒	The exact sample size (*n*) for each experimental group/condition, given as a discrete number and unit of measurement
☐	☒	A statement on whether measurements were taken from distinct samples or whether the same sample was measured repeatedly
☐	☒	The statistical test(s) used AND whether they are one- or two-sided<br>*Only common tests should be described solely by name; describe more complex techniques in the Methods section.*
☒	☐	A description of all covariates tested
☒	☐	A description of any assumptions or corrections, such as tests of normality and adjustment for multiple comparisons
☐	☒	A full description of the statistical parameters including central tendency (e.g. means) or other basic estimates (e.g. regression coefficient) AND variation (e.g. standard deviation) or associated estimates of uncertainty (e.g. confidence intervals)
☐	☒	For null hypothesis testing, the test statistic (e.g. *F*, *t*, *r*) with confidence intervals, effect sizes, degrees of freedom and *P* value noted<br>*Give P values as exact values whenever suitable.*
☒	☐	For Bayesian analysis, information on the choice of priors and Markov chain Monte Carlo settings
☒	☐	For hierarchical and complex designs, identification of the appropriate level for tests and full reporting of outcomes
☐	☒	Estimates of effect sizes (e.g. Cohen's *d*, Pearson's *r*), indicating how they were calculated

Our web collection on statistics for biologists contains articles on many of the points above.

Software and code

Policy information about availability of computer code

Data collection	No software was used
Data analysis	GraphPad Prism software v8.0.2<br>Fiji v 1.53c<br>Snakemake v7.6.1<br>Trimmomatic v0.39<br>Bowtie2 v2.2.5<br>deepTools v3.5.1<br>MACS2 v2.2.6<br>Samtools v1.9<br>Juicer v1.6<br>Hi-C Explorer v3.7.2<br>fanc v0.9.23<br>Cellranger-arc v2.0.0<br>ArchR v1.0.1<br>Seurat v4.3.0<br>Deseq2 v1.38.3<br>Picard tools 2.26.9<br>Bedtools v2.30.0<br>ROSE 3<br>Rstudio Server v2022.07.1

IGV v2.13.1
Juicebox v1.11.08
bigWigMerge v2
bedGraphToBigWig v4
The origninal code used for the data processing of this work is available at: https://github.com/edobelini/Sessa-Lab/tree/main/Balanced_SET_levels_favor_the_correct_enhancer_repertoire_during_cell_fate_acquisition or https://zenodo.org/badge/latestdoi/619963042

For manuscripts utilizing custom algorithms or software that are central to the research but not yet described in published literature, software must be made available to editors and reviewers. We strongly encourage code deposition in a community repository (e.g. GitHub). See the Nature Portfolio guidelines for submitting code & software for further information.

Data

Policy information about availability of data

All manuscripts must include a data availability statement. This statement should provide the following information, where applicable:

- Accession codes, unique identifiers, or web links for publicly available datasets
- A description of any restrictions on data availability
- For clinical datasets or third party data, please ensure that the statement adheres to our policy

The ATAC-seq, ChIP-seq, Hi-C, RNA-seq and scMultiome data generated in this study have been deposited in the Gene Expression Omnibus (GEO) database in the are available at GSE212252. NPCs RNA-seq data are available at GSE171266. Control PSC ATAC-seq were obtained from GSE108248 https://docs.github.com/en/repositories/archiving-a-github-repository/referencing-and-citing-content. NPCs and NPC-derived neurons Hi-C control dataset54 used for this publication were obtained from NIMH Repository & Genomics Resource, a centralized national biorepository for genetic studies of psychiatric disorders. The raw number associated with bar plots pertaining the associated figures, ATAC-seq peaks, ChIP-seq peaks, differentially expressed genes, RNA normalized counts, single cells matrices, functional enrichment results generated in the study are available in the source data and supplementary information files.

Human research participants

Policy information about studies involving human research participants and Sex and Gender in Research.

Reporting on sex and gender	*Use the terms sex (biological attribute) and gender (shaped by social and cultural circumstances) carefully in order to avoid confusing both terms. Indicate if findings apply to only one sex or gender; describe whether sex and gender were considered in study design whether sex and/or gender was determined based on self-reporting or assigned and methods used. Provide in the source data disaggregated sex and gender data where this information has been collected, and consent has been obtained for sharing of individual-level data; provide overall numbers in this Reporting Summary. Please state if this information has not been collected. Report sex- and gender-based analyses where performed, justify reasons for lack of sex- and gender-based analysis.*
Population characteristics	*Describe the covariate-relevant population characteristics of the human research participants (e.g. age, genotypic information, past and current diagnosis and treatment categories). If you filled out the behavioural & social sciences study design questions and have nothing to add here, write "See above."*
Recruitment	*Describe how participants were recruited. Outline any potential self-selection bias or other biases that may be present and how these are likely to impact results.*
Ethics oversight	*Identify the organization(s) that approved the study protocol.*

Note that full information on the approval of the study protocol must also be provided in the manuscript.

Field-specific reporting

Please select the one below that is the best fit for your research. If you are not sure, read the appropriate sections before making your selection.

☒ Life sciences ☐ Behavioural & social sciences ☐ Ecological, evolutionary & environmental sciences

For a reference copy of the document with all sections, see nature.com/documents/nr-reporting-summary-flat.pdf

Life sciences study design

All studies must disclose on these points even when the disclosure is negative.

Sample size	No statistical methods were used to pre-determine sample sizes, but our sample sizes are similar to those reported in previous publications (see De la torre Ubieta et al., 2018; Rajarajan et al., 2018; Conforti P. et al., 2020; Inak G. et al., 2021; Xie Y. et al., 2020; for experiments concerning isogenic cell line derivation, PSCs characterization and 2D differentiation. See Sessa et al., 2019,La Manno et al.,2018 for mous model experiments). We used independent isogenic control cell lines and singenic lettermates for animal experiments to increase the robustness of the results. However, sample size per group and condition is equal to or greater than generally accepted standard of three biological replications per group.

Data exclusions	No data were excluded
Replication	We repeated all experiments using at least three biological replicates over distinct independent experiments. We specified the number of biological replicates and independent experiments in the respective figure legends.
Randomization	We plated the cells in a random distribution onto cell culture and multi-well plate positions, and randomly assigned them to experimental groups. We perfomed cell counting on random miscroscope view fields. Animals were randomly selected for experimental analysis from different litter with appropriate genotype. Covariates like sex were not relevant for this kind of experiments
Blinding	Data collection and analyses were not performed blind to the conditions due to obvious differences between groups. The same results have been repeated by multiple members of the research team.

Reporting for specific materials, systems and methods

We require information from authors about some types of materials, experimental systems and methods used in many studies. Here, indicate whether each material, system or method listed is relevant to your study. If you are not sure if a list item applies to your research, read the appropriate section before selecting a response.

Materials & experimental systems

n/a	Involved in the study
☐	☒ Antibodies
☐	☒ Eukaryotic cell lines
☒	☐ Palaeontology and archaeology
☐	☒ Animals and other organisms
☒	☐ Clinical data
☒	☐ Dual use research of concern

Methods

n/a	Involved in the study
☐	☒ ChIP-seq
☐	☒ Flow cytometry
☒	☐ MRI-based neuroimaging

Antibodies

Antibodies used

The antibodies used in this work are listed with the details (producer, species or origin, dilution of usage, and RRID) in the Supplementary Table N° 1.

Validation

The antibodies used in this work are all commercial and widely tested in literature for the proposed assay.

Mouse monoclonal anti-SOX2 (Clone # 245610): R&D system, Cat# MAB2018, RRID:AB_358009
Generation of the human induced pluripotent stem cell (hiPSC) line PSMi006-A from a patient affected by an autosomal recessive form of long QT syndrome type 1
Manuela Mura et al., 2019. DOI: 10.1385/1-59745-046-4:91

Rabbit polyclonal anti-Calnexin: Sigma-Aldrich, Cat#C4731, RRID:AB_476845
PI4KB on Inclusion Bodies Formed by ER Membrane Remodeling Facilitates Replication of Human Parainfluenza Virus Type 3
Zhifei Li et al., 2019. DOI: 10.1016/j.celrep.2019.10.052

Chicken polyclonal anti-GFP: Thermo Fisher Scientific, Cat#A10262, RRID:AB_2534023
Maturation of spinal motor neurons derived from human embryonic stem cells
Tomonori Takazawa et al., 2012. DOI: 10.1371/journal.pone.0040154

Rabbit monoclonal anti-SET/TAF-I (clone EPR12973): Abcam, Cat#ab181990, RRID:AB_2737445
A Membraneless Organelle Associated with the Endoplasmic Reticulum Enables 3'UTR-Mediated Protein-Protein Interactions
Weirui Ma and Christine Mayr, 2018. DOI: 10.1016/j.cell.2018.10.007

Rabbit polyclonal anti-Setbp1: Proteintech, Cat#16841-1-AP, RRID:AB_2185750
Somatic SETBP1 mutations in myeloid malignancies
Hideki Makishima et al., 2013. DOI: 10.1038/ng.2696

AAnti-Doublecortin antibody: Abcam, Cat#ab18723, RRID: AB_732011
In vitro human stem cell derived cultures to monitor calcium signaling in neuronal development and function
Yojet Sharma et al., 2020. DOI: 10.12688/wellcomeopenres.15626.1

Rabbit polyclonal anti-Histone H3: Abcam, Cat# ab1791, RRID:AB_302613
The integrated stress response induces R-loops and hinders replication fork progression
Josephine Ann Mun Yee Choo et al., 2020. DOI: 10.1038/s41419-020-2727-2

Rabbit Anti-Histone H3 (acetyl K27) antibody: Abcam, Cat# ab4729, RRID:AB_2118291
Chromatin profiling reveals relocalization of lysine-specific demethylase 1 by an oncogenic fusion protein
Emily R Theisen et al., 2021, DOI: 10.1080/15592294.2020.1805678

Rabbit Anti-Histone H3 (acetyl K27) antibody: Diagenode, Cat# C15410196, RRID:AB_2637079

Epigenomic analysis reveals a dynamic and context-specific macrophage enhancer landscape associated with innate immune activation and tolerance
Phing Zhang et al. 2022, DOI:https://doi.org/10.1186/s13059-022-02702-1

Rabbit Anti-Histone H3 (acetyl K9 + K14 + K18 + K23 + K27) antibody: Abcam, Cat#ab47915, RRID:AB_873860
Regional identity of human neural stem cells determines oncogenic responses to histone H3.3 mutants
Raul Bardini Bressan et al., 2022, DOI:10.1016/j.stem.2021.01.016

Rabbit Recombinant Anti-Histone H3 (acetyl K9) antibody [Y28]: Abcam, Cat#ab32129, RRID:AB_732920
Targeting the CoREST complex with dual histone deacetylase and demethylase inhibitors
Jay H Kalin et al., 2018, DOI: 10.1038/s41467-017-02242-4

Rabbit Recombinant Anti-Histone H4 (acetyl K5) antibody [EP1000Y]: Abcam, Cat#ab51997, RRID:AB_2264109
Genome-wide occupancy reveals the localization of H1T2 (H1fnt) to repeat regions and a subset of transcriptionally active chromatin domains in rat spermatids
Vasantha Shalini et al., 2021, DOI:Vasantha Shalini

Rabbit Anti-TBR2 / Eomes antibody: Abcam, Cat#ab23345, RRID:AB_778267
Capture of Mouse and Human Stem Cells with Features of Formative Pluripotency
Masaki Kinoshita et al., 2021, DOI:10.1016/j.stem.2020.11.005

Rabbit GFP antibody: Thermo-Fisher Scientific, Cat#A11122, RRID:AB_221569
A novel Wnt5a-Frizzled4 signaling pathway mediates activity-independent dendrite morphogenesis via the distal PDZ motif of Frizzled 4
Wen-Jie Bian et al., 2015, DOI:10.1002/dneu.22250

Rabbit anti-RFP antibody: MBL International, Cat# PM005, RRID:AB_591279
Ecd promotes U5 snRNP maturation and Prp8 stability
Steffen Erkelenz et al., 2021, DOI:10.1093/nar/gkaa1274

Mouse V5 Tag Monoclonal antibody: Thermo-Fisher Scientific, Cat#R960-25. RRID:AB_2556564
Proteomic Analysis of Unbounded Cellular Compartments: Synaptic Clefts
Ken H Loh et al., 2016, DOI:10.1016/j.cell.2016.07.041

Mouse/Rat CD31/PECAM-1 antibody: R and D Systems, Cat# AF3628, RRID:AB_2161028
Suprachiasmatic nucleus-mediated glucose entry into the arcuate nucleus determines the daily rhythm in blood glycemia
Betty Rodríguez-Cortés et al., 2022, DOI:10.1016/j.cub.2021.12.039

Mouse TER-119/Erythroid Cells antybody: BioLegend, Cat# 116241, RRID:AB_2563789
Stress-Induced Changes in Bone Marrow Stromal Cell Populations Revealed through Single-Cell Protein Expression Mapping
Nicolas Severe et al., 2019, DOI:10.1016/j.stem.2019.06.003

PE Mouse CD71 anotibody: BioLegend, Cat# 113808, RRID:AB_313569
C/EBPα and GATA-2 Mutations Induce Bilineage Acute Erythroid Leukemia through Transformation of a Neomorphic Neutrophil-Erythroid Progenitor
Cristina Di Genua et al., 2020, DOI: 10.1016/j.ccell.2020.03.022

PE/Cyanine5 Mouse TER-119/Erythroid Cells antibody: BioLegend, Cat# 116210, RRID:AB_313711
Ontogenic shifts in cellular fate are linked to proteotype changes in lineage-biased hematopoietic progenitor cells
Maria Jassinskaja et al., 2021, DOI: 10.1016/j.celrep.2021.108894

Eukaryotic cell lines

Policy information about cell lines and Sex and Gender in Research

Cell line source(s)	SGS patients IPSCs from previous work were used (Banfi et al., 2021, DOI:https://doi.org/10.1038/s41467-021-24391-3) . IPSCs were derived from fibroblast gifted from from Dr. Alexander Hoischen (Radboud umc, the Nederlands).
Authentication	IPSCs were routinely tested for mycoplasma, stemness and pluripotency, Karyotype and for the presence of expected mutations (Sanger sequencing).
Mycoplasma contamination	all cell lines were negative for mycoplama contamination (PCR-test)
Commonly misidentified lines (See ICLAC register)	No commonly misidentified cell lines were used.

Animals and other research organisms

Policy information about studies involving animals; ARRIVE guidelines recommended for reporting animal research, and Sex and Gender in Research

Laboratory animals	C57Black6j strain for breedings. Transgenic lines, CMV::CRE, Nestin:::CRE and R2626LSL-SETBP1 KI all in C57 Black6j background. Mice were maintained were maintained at the San Raffaele Scientific Institute institutional facility in a pathogen-free environment. Temperature and air flow were controlled and constant (T = 22°+/-2; RH = 55% +/-5). Light cycle of 12h phase was used (not inverted).<br>Age used: animals of the pure lines and double transgenic were used for crossing in adult ages (2-6 months of ages). Experimental animals were analyzed at: a) embryonic stage Embryonic day 9.5 (E.9.5) and E10.5 (constitutive mutants) with investigation also at E12.5, E14.5 and P0 (with no mutants found); b) E14.5, P2 and P30 the brain specific mutants.<br>Zebrafish (Danio rerio) embryos were obtained from natural matings of the Wild-type strain AB and raised in E3 medium at 28.5°C on a 14/10-hour light/dark cycle. All experimental procedures were carried out at the San Raffaele Scientific Institute Institutional facility and performed in accordance with experimental protocols approved by local Institutional Animal Care and Use Committees (IACUC).
Wild animals	No wild animals were used in this study
Reporting on sex	No sex based analysis were performed.
Field-collected samples	No field-collected samples were used in this study
Ethics oversight	Experiments were performed in accordance with experimental protocols approved by local Institutional Animal Care and Use Committees (IACUC) at Ospedale San Raffaele. For mice: IACUC #1182 (ministerial authorization 381/2021-PR) – IACUC #1264 (ministerial authorization 261/2022-PR). For zebrafish: Aut. Prot, 102093; Aut. Min, 06/2021-UT

Note that full information on the approval of the study protocol must also be provided in the manuscript.

ChIP-seq

Data deposition

☒ Confirm that both raw and final processed data have been deposited in a public database such as GEO.

☒ Confirm that you have deposited or provided access to graph files (e.g. BED files) for the called peaks.

Data access links<br>*May remain private before publication.*	https://www.ncbi.nlm.nih.gov/geo/query/acc.cgi?acc=GSE212252
Files in database submission	NPCD868D_Chip_H3K27ac_1_R1_001.fastq.gz, NPCD868D_Chip_H3K27ac_1_R2_001.fastq.gz,<br>NPCD868D_Chip_H3K27ac_2_R1_001.fastq.gz, NPCD868D_Chip_H3K27ac_2_R2_001.fastq.gz,<br>NPCD868D_Chip_SET_1_R1_001.fastq.gz, NPCD868D_Chip_SET_1_R2_001.fastq.gz,<br>NPCD868D_Chip_SET_2_R1_001.fastq.gz, NPCD868D_Chip_SET_2_R2_001.fastq.gz,<br>NPCD868D_Chip_Input_R1_001.fastq.gz, NPCD868D_Chip_Input_R2_001.fastq.gz,<br>NPCD868N_Chip_H3K27ac_1_R1_001.fastq.gz,NPCD868N_Chip_H3K27ac_1_R2_001.fastq.gz,<br>NPCD868N_Chip_H3K27ac_2_R1_001.fastq.gz, NPCD868N_Chip_H3K27ac_2_R2_001.fastq.gz,<br>NPCD868N_Chip_SET_1_R1_001.fastq.gz, NPCD868N_Chip_SET_1_R2_001.fastq.gz,<br>NPCD868N_Chip_SET_2_R1_001.fastq.gz, NPCD868N_Chip_SET_2_R2_001.fastq.gz,<br>NPCD868N_Chip_Input_R1_001.fastq.gz, NPCD868N_Chip_Input_R2_001.fastq.gz,<br>NPCD868D_Chip_H3K27ac_1_narrowPeak, NPCD868D_Chip_H3K27ac_2_narrowPeak,<br>NPCD868D_Chip_SET_1_peaks.broadPeak, NPCD868N_Chip_H3K27ac_1_narrowPeak,<br>NPCD868N_Chip_H3K27ac_2_narrowPeak, NPCD868N_Chip_SET_1_peaks.broadPeak,<br>NPCD868N_Chip_SET_2_peaks.broadPeak<br>NPCD868D_Chip_SET_2_peaks.broadPeak
Genome browser session<br>(e.g. UCSC)	N/A

Methodology

Replicates	We performed 2 techincal replicates for each experimental condition (Affected and control condition).
Sequencing depth	30 milion paired-end reads.
Antibodies	Rabbit Anti-Histone H3 (acetyl K27) antibody: Diagenode, Cat# C15410196, RRID:AB_2637079<br>Epigenomic analysis reveals a dynamic and context-specific macrophage enhancer landscape associated with innate immune activation and tolerance<br>Phing Zhang et al. 2022, DOI:https://doi.org/10.1186/s13059-022-02702-1<br><br>Rabbit monoclonal anti-SET/TAF-I (clone EPR12973): Abcam, Cat#ab181990, RRID:AB_2737445<br>A Membraneless Organelle Associated with the Endoplasmic Reticulum Enables 3'UTR-Mediated Protein-Protein Interactions<br>Weirui Ma and Christine Mayr, 2018. DOI: 10.1016/j.cell.2018.10.007

Peak calling parameters	macs2 callpeak -t chip.bam -c input.bam-f BAMPE --nomodel --qvalue 0.01 --keep-dup all --call-summits
Data quality	All epigenomics dataset were processed using a custom Snakemake (v7.6.1) pipeline, from reads adaptor trimming up to peak calling and normalized track generation. FASTQ were first quality checked to evaluate sequence output with using FastQC (Andrews, S. FastQC A Quality Control tool for High Throughput Sequence Data). Reads were trimmed using Trimmomatic (v0.39) and then aligned to reference genome hg38 using Bowtie2 using the --very-sensitive option. Non canonical and M chromosomes were removed from Bam files using Samtools (v1.9) and Picard tools ("Picard Toolkit." 2019. Broad Institute, GitHub Repository. https://broadinstitute.github.io/picard/) was used to remove PCR optical duplicates, before proceeding with further analysis. Normalized BigWig files for genomics track visualization and for subsequent analysis were generated using deepTools (v3.5.1)92 'bamCoverage with the following parameters --normalizeUsing RPKM --binSize 10 --smoothLength 300 –effectiveGenomeSize --ignoreDuplicates --skipNAs –exactScaling'. To obtain a single merged tracks from single experimental replicates for each experimental condition UCSC bigWigMerge was used. Peak calling was performed using MACS2 (v2.2.6) from preprocessed Bam files for each experimental replicate from each condition.<br>Blacklist peaks for hg38 was used to filter off-targets regions. A total of 68303 peaks above FDR 1 were obtained in the control condition and 65899 were obtained in the mutant condition.
Software	MACS2 v2.2.6

Flow Cytometry

Plots

Confirm that:

☒ The axis labels state the marker and fluorochrome used (e.g. CD4-FITC).

☒ The axis scales are clearly visible. Include numbers along axes only for bottom left plot of group (a 'group' is an analysis of identical markers).

☒ All plots are contour plots with outliers or pseudocolor plots.

☒ A numerical value for number of cells or percentage (with statistics) is provided.

Methodology

Sample preparation	Single-cell suspensions were prepared from Rosa26::LoxP-STOP-LoxP-hSETBP1G870S/+ (WT) and Rosa26::hSETBP1G870S/+ (full mutant) embryos YS. After collagenase digestion, cells were resuspended in Calcium Magnesium-Free PBS, FBS 10%, Penicillin-Streptomycin 1%, EDTA 2mM and stained with Rat anti-mouse CD71-PE (BioLegend Cat# 113808, RRID:AB_313569), Rat anti-mouse Ter119 -PE-Cy5 (BioLegend Cat# 116210, RRID:AB_313711).
Instrument	BD LSR-Fortessa X-20 Cytometer
Software	FlowJo software v10.8.1
Cell population abundance	Abundance of cell population was calculated after prefitering of live cells. See bar grah in supplementary fig.8 d and supplementary fig.8 e for gating strategy
Gating strategy	Gates were set unstained, single stained and fluorescence-minus-one (FMO) control

☒ Tick this box to confirm that a figure exemplifying the gating strategy is provided in the Supplementary Information.
